# Acute toxicity assessment of polyaniline/Ag nanoparticles/graphene oxide quantum dots on *Cypridopsis vidua* and *Artemia salina*

**DOI:** 10.1038/s41598-021-84903-5

**Published:** 2021-03-05

**Authors:** Azza Shokry, Marwa Khalil, Hesham Ibrahim, Moataz Soliman, Shaker Ebrahim

**Affiliations:** 1 Department of Environmental Studies, Institute of Graduate Studies and Research, Alexandria University, P.O. Box 832, Alexandria, Egypt; 2 Department of Nanotechnology and Composite Materials, Institute of New Materials and Advanced Technology, City of Scientific Research and Technological Applications (SRTA-City), New Borg El Arab City, P.O. Box 21934, Alexandria, Egypt; 3 Department of Materials Science, Institute of Graduate Studies and Research, Alexandria University, P.O. Box 832, Alexandria, Egypt

**Keywords:** Environmental sciences, Chemistry, Nanoscience and technology

## Abstract

Nanotoxicology is argued and considered one of the emerging topics. In this study, polyaniline (PANI)/2-acrylamido-2-methylpropanesulfonic acid (AMPSA) capped silver nanoparticles (NPs)/graphene oxide (GO) quantum dots (QDs) nanocomposite (PANI/Ag (AMPSA)/GO QDs NC) as a nanoadsorbent has a potential for removal of toxic hexavalent chromium (Cr(VI)) ions from water. The acute toxicity of this NC was evaluated on *Artemia salina* and freshwater *Ostracods* (*Cypridopsis vidua)* larvae for 48 h. The measurements were made at 24 and 48 h with 3 repetitions. The 50% effective concentration (EC_50_) values of the NC were determined after the exposure of these organisms. According to the results of the optical microscope, it was found that both experimental organisms intake the NC. In the toxicity results of *Ostracods*, the NC had a highly toxic effect only at 250 mg/L after 48 h and the EC_50_ value was 157.6 ± 6.4 mg/L. For *Artemia salina* individuals, it was noted that they were less sensitive than the *Ostracods* and EC_50_ value was 476 ± 25.1 mg/L after 48 h. These results indicated that PANI/Ag (AMPSA)/GO QDs NC has low toxicity towards both investigated organisms.

## Introduction

Engineered nanoparticles are defined as a material has a particle size ranged from 1 to 100 nm^1^. Nanoscience recently plays an important role by providing new materials with unique characteristics for remediation/treatment and sensing applications^2–6^. The research field called “nanoecotoxicology” explores the impacts of nanoparticles on the organisms and their ecosystems. This field has widely studied since some of the engineered nanoparticles reach water resources^7^.

Carbon nanostructures such as graphene, graphene oxide and carbon nanotubes have unique physical and chemical properties with wide potential applications in various fields^8–14^. Quantum dots (QDs) as colloidal nanocrystals have unique photophysical, electronic and chemical properties due to their large absorption coefficients, high emission and high photostability^15^. Carbon dots are a new type of carbon-based material class and their low-cost and easy preparation are resulting in their innumerable applications^16,17^. The green methods are encouraged to synthesize carbon dots because they produce harmless products. In addition, graphene oxide quantum dots (GO QDs) with a particle size ranging from 2 to 20 nm are attracted due to their chemical inertness, low toxicity, biocompatibility, eco-friendly, and good solubility^18^. It was reported that the 50% effective concentration (EC_50_) value of GO for *Artemia salina* is 368.18 mg/L^19^.

Silver nanoparticles (Ag NPs) have anti-microbial effect, high chemical stability, interested optical and electronic properties, high surface reactivity to enhance the adsorption capabilities and high surface to volume ratio^20–22^. However, Juganson et al. reported that Ag NPs have the highest toxicity among other nanomaterials^23^. Several studies investigated the toxicity of Ag NPs on *Artemia salina* and determined the values of EC_50_ after 24 h and 72 h to be 19.63 mg/L and 10.70 ± 1.3 mg/L, respectively^24,25^.

Polyaniline (PANI) and its nanocomposites (NCs) with high stability, high electroactivity, high conductivity and low cost are candidate to apply in the fields of sensors^6,26–29^ , energy storage^10^ and water treatment^5,30–32^. Yslas et al. reported that there is no toxicological effect of PANI nanofibers for *Rhinella arenarum* larvae exposed to PANI even at the highest concentration, 400 mg/L^33^. Ibarra et al. confirmed that the 50% lethal concentration (LC_50_) values of PANI NPs dispersed in polyvinylpyrrolidone and polyN-isopropylacrilamide on the larvae of *Rhinella arenarum* were 1500 mg/L and 1170 mg/L, respectively^34^.

Chromium compounds are one of the highest toxic materials and consider as environmental pollutants. High levels of these compounds affect cellular structures. Therefore, there are great efforts to develop selective sensors and adsorbents for chromium with various nanomaterials, nanocomposites or hybrids materials^5,32,35–38^.

Bioassay tests are important for determining the applicability of the synthesized materials in water treatment. The exposure to the nanomaterials and their possible effects should be evaluated in freshwater and saltwater. *Cypridopsis vidua*, also called *Cypridopsis* Müller are freshwater *Ostracode*. The most dominant species of freshwater *Ostracods* in Egypt is *Cypridopsis vidua*^39^. *Ostracods* have been used as one of freshwater crustaceans in the ecotoxicological studies and also for toxicity monitoring of water, soil and river sediment^40–42^.

*Artemia salina* is another crustacean from salt lakes and hypersaline ecosystems. *Artemia salina* is also considered as one of the test species by the United States Environmental Protection Agency (US-EPA) for acute toxicity testing^43^. Many studies have used *Artemia salina* as a model organism to study the ecotoxicity of some nanoparticles^19,25,44–46^.

The current work is engaged with testing of PANI/2-acrylamido-2-methylpropanesulfonic acid capped Ag NPs/GO QDs (PANI/Ag (AMPSA)/GO QDs) toxicity which have been prepared and used in the detection and removal of toxic hexavalent chromium (Cr(VI)) from polluted water^6,32,47^. This test is conducted on larvae of freshwater *Ostracode* (*Cypridopsis vidua*) and saltwater *Artemia salina*. The acute toxicity of different concentrations of PANI/Ag (AMPSA)/GO QDs is studied for 24 h and 48 h of exposure. Also, the uptake and accumulation of the NC in the *Ostracode and Artemia* larvae are observed and discussed after 24 h of exposure.

## Materials and methods

### Preparation and characterization techniques

GO QDs were synthesized by direct glucose (BDH Prolabo Chemicals) pyrolysis and dodecylbenzene sulfonic acid (El-Gomhoria Chemical Company, Egypt) doped PANI was synthesized by chemical oxidative polymerization method, respectively. An amount of glucose (2 g) was put in a beaker and heated to 250 °C for 20 min and the orange liquid was added drop by drop to 100 mL of 12.5% NH_3_ solution with stirring. This solution was heated at 70 °C for 3 h. 0.03 mL of aniline monomer (99.0%, Research Lab, India) was added to 10 mL deionized water and mixed with 10 mL of water (0.3 g DBSA and 0.1 g APS) through 1 h. Ag (AMPSA) NPs were prepared by the chemical reduction of silver nitrate (99.8%, PRS Panreac, Spain) using sodium borohydride (99.0%, Merck, Germany) as a reducing agent. 1.2 mL 10 mM sodium borohydride was inserted to 36.8 mL of deionized water in ice bath with stirring. Then, 0.4 mL 10 mM AgNO_3_ was mixed and added dropwise and 0.3 mL 10 mM AMPSA (97.0%, Acros Organics, Germany) was added dropwise to the mixture with stirring for 10 min. PANI/Ag (AMPSA) NC was prepared by in situ oxidative polymerization of aniline in with Ag (AMPSA) NPs. Aniline (0.03 mL) was dissolved in 10 mL previously prepared Ag (AMPSA) NPs^47^.

In addition, PANI/Ag (AMPSA)/GO QDs NC was prepared by in situ oxidative polymerization of aniline in presence of the nanoparticles. PANI/Ag (AMPSA)/GO QDs NC was prepared with the same procedure PANI/Ag (AMPSA) NC was prepared as above. The ternary NC was prepared by mixing 10 mL of AMPSA capped Ag NPs and 1 mL of the previously prepared GO QDs solution under magnetic stirring for 10 min^47^.

All details regarding the synthesis and characterization of PANI/Ag (AMPSA)/ GO QDs NC and its related materials in the present study can be found in our recent publications^32,47^. From our previous work^47^, it was noted that PANI/Ag (AMPSA)/GO QDs NC has the highest and sharpest PL intensity compared with each component of the NC of PANI, GO QDs and Ag (AMPSA) NPs. Therefore, PANI/Ag (AMPSA)/GO QDs NC was selected for Cr(VI) detection. PANI/Ag (AMPSA)/GO QDs NC was used as a sensitive fluorescence quenching probe for detecting Cr(VI). The explanation of the quenching mechanism of this NC is based on the synergistic effect of inner filter effect, the ground state compounds formation and ions exchange^6^.

### Test organisms

The freshwater *Ostracod was* obtained from a local ornamental fish shop and was identified as *Cypridopsis vidua*. The identification of species was determined by the National Institute for Oceanography and Fisheries, Alexandria, Egypt. The *Ostracods* were reared in-house in 500 mL glass Jar at 28 ± 2 °C and fed with yeast powder and algae-containing water under the natural sunlight. The moderately hard synthetic freshwater (96 mg/L NaHCO_3_ (99%, Acros Organics, Germany), 60 mg/L Ca(NO_3_)_2_. 4H_2_O (99%, PRS Panreac, Spain), 123 mg/L MgSO_4_·7H_2_O (99.0%, Fisher Scientific, UK) and 4 mg/L KNO_3_ (98%, Fisher Scientific, UK)) was used as a test medium^48^. Adults of *Ostracod* were isolated in 50 mL beakers and allowed to produce offspring and the young *Ostracods* (neonates) produced were used in the experiments.

Commercially available dehydrated cysts of *Artemia salina* (origin: salt lake, U.S.) were obtained from Sera, Germany. *Artemia* life cycle begins as cysts, then emerged embryos, nauplii, finally larvae and adults. The hatching procedure followed the ARC-Test method^49^. The artificial seawater of 35 g/L was used for the hatching as well as a testing solution^50^.

### Acute toxicity tests

Toxicity evaluation of PANI/Ag (AMPSA)/GO QDs NC was undertaken by determining the EC_50_ for both of *Artemia salina* and freshwater *Ostracod (Cypridopsis vidua*) and using an immobilization process as an acute endpoint. Forty-eight hour acute toxicity test on *Artemia salina* was performed according to ISO/TS 20787 standard operating procedure with a slightly modification^51^. For *Cypridopsis vidua* we used a modified version test of the OSTRACODTOXKIT F^52^. Two samples of PANI/Ag (AMPSA)/GO QDs NC (25 mg) were suspended in 25 mL of *Ostracods* or *Artemia* culture media to prepare two stock concentrations of 1000 mg/L. The two samples were then suspended and homogenized using an ultrasonic bath (Focus, Spain). Concentrations of 10, 50, 150, 250, 350, 450, 550 and 1000 mg/L of PANI/Ag (AMPSA)/GO QDs NC were tested to determine their acute toxicity on the *Ostracods* (24 h old) larva and *Artemia* (instar ӀӀ–ӀӀӀ) stages. Sorgeloos et al.^53^ reported that in the first *Artemia* larval stage (instar I), the digestive tract of the nauplius is not in contact yet with the external medium, and the larva only consumes its yolk. In the second larval stage (instar II), it starts the feeding on particulate matter. And, instar I larvae are significantly more resistant to chromic acid than instar II. So, they recommended that bioassays with *Artemia* larvae should be carried out as short-term toxicity tests with instar ӀӀ-ӀӀӀ stages^53^.

Groups of 10 *Artemia* (instar ӀӀ-ӀӀӀ) stages and *Ostracods* larvae were placed in wells (flat bottom with lid, pre-sterilized, Costar, US) contained 10 mL of the nanocomposite suspension. During the exposure, the tested larvae were not fed and kept under room temperature (28 °C). After 24 and 48 h, the numbers of immobilized larvae (completely motionless) were counted under a binocular microscope (PZ0, Poland) and the immobilization of each treatment was calculated. Immobilization percentage was calculated according to the following equation^54^:

1 $$Immobilization \; \%= \frac{immobilized \; larvae}{Total \;  larvae} \times 100$$

Negative controls exposure without PANI/Ag (AMPSA)/GO QDs NC were performed in parallel for both the test organisms and in the positive controls, solution of 50 mg/L potassium dichromate (99.0%, Merck, Germany) was used according to the ISO/TS 20787 method^51^. *Ostracod*s culture and *Artemia* hatching media containing the same number of organisms (ten organisms) were used. Results were recorded and ensured that the percentage of immobilization in negative control did not exceed 10%. Acute toxicity test was conducted in triplicates and EC_50_ values were calculated using the probit method of analysis in SPSS version 23 software.

### Toxicity testing of the Cr(VI) treated water

The toxic effect of three different concentrations of Cr(VI) solutions (10, 30 and 60 mg/L) before and after treatment with 1 g/L of PANI/Ag (AMPSA)/GO QDs NC was investigated using 48 h acute toxicity bioassays expressed in immobilization percentage (%) for both *Ostracod* and *Artemia salina*. The Cr(VI) removal experiments and analyses methods using UV–Visible spectrophotometer (Evolution 300, Thermo scientific, USA) are reported in our previous publication^32^.

### Uptake of PANI/Ag (AMPSA)/GO QDs NC by *Ostracods* and *Artemia salina*

The uptake and accumulation of PANI/Ag (AMPSA)/GO QDs NC by the *Ostracod*s and *Artemia salina* were observed using an optical microscope (ZEISS Primo Star, Germany), and images were taken by an integrated digital camera (ZEISS, Germany).

## Results and discussion

### Acute toxicity of PANI/Ag (AMPSA)/GO QDs NC on the *Ostracods*

Table 1 shows the *Ostracods* mortality with different concentrations of the nanocomposite and the corresponding EC_50_. In addition, the toxicity unit (TU) values obtained after 24 and 48 h of exposure are depicted in Table 2. TU is equal to 100/EC_50_, which is the reciprocal of the concentration that causes 50% of the organisms to immobilize by the end of the acute exposure period. Results obtained from Table 1 indicate that the exposure to 10 mg/L of PANI/Ag (AMPSA)/GO QDs has not any toxic effects on the *Ostracods*. It is noted that further increasing of the NC concentration raises the immobilization. After 24 and 48 h of exposure, 100% immobilization is observed at concentration higher than 450 mg/L NC. Ag NPs and GO QDs dissolution and their aggregation are assumed to control the toxicity. The release of dissolved Ag^+^ ions and GO from the NC surface to the media can be responsible for the toxicity^55^.

**Table 1:** *Ostracods* mean immobilization % after 24 and 48 h of exposure to different concentrations of PANI/Ag (AMPSA)/GO QDs NC.

Concentration (mg/L)	24 h immobilization %	48 h immobilization %
10	0	0
50	7	10
150	37	43
250	60	70
350	73	80
450	87	93
550	100	100
1000	100	100

**Table 2:** EC_50_ and TU of PANI/Ag (AMPSA)/GO QDs NC after 24 and 48 h of exposure to *Ostracods.*

EC_50_	Mean values (mg/L)	Toxicity level	95% confidence limits (mg/L)
EC_50_ at 24 h	187.1 ± 15.7	Not acutely toxic	121.599–251.037
EC_50_ at 48 h	157.6 ± 6.4	(EC_50_ ˃100)	99.361–213.655
TU at 24 h	0.53	Low toxic	
TU at 48 h	0.63	(TU˂1)	

The results in Table 2 display that the 48 h EC_50_ value is lesser than 24 h EC_50_. The mean values of EC_50_ and TU for PANI/Ag (AMPSA)/GO QDs after 24 and 48 h are (187.1 and 157.6 mg/L) and (0.53 and 0.63), respectively. Based on the general criteria proposed by Canton et al. and Tonkes et al. for the acute toxicity classification of effluent, PANI/Ag (AMPSA)/GO QDs NC is not acutely toxic (EC_50_ ˃100 mg/L) and with respect to TU is low toxic (TU˂1)^56,57^.

### Immobilization values are the mean values of three replicates ± standard deviation

Ag NPs have a toxic effect on aquatic organisms and this toxicity is highly depending on the particle size and different surface coatings. Several publications reported that polymer-coated Ag NPs significantly decreased the toxicity of the bare or citrate-coated Ag NPs. Moreover, they found that surface coating was the major factor that determines the toxicity compared with particle size^58–60^. On the other hand, it was found that there is no acute toxicity for the graphene derivatives demonstrated on the crustaceans although the optical microscope images showed the presence of these graphene derivatives aggregated in the gut^19,61^.

The main acute toxicity mechanisms governed the toxicity of the nanoparticles in aquatic organisms are the ion regulatory disturbance and competitive inhibition of K or Na ion-dependent adenosine triphosphate (Na^+^, K^+^-ATPase) activity^62–64^. For aquatic organisms in general, including invertebrates, there are very few studies which have addressed this issue. Meanwhile, they reported that after penetrating these NPs into the living cells, it produced reactive oxygen species and induced toxic effects such as membrane lipid peroxidation, mitochondrial damage, DNA damage, and consequently cell apoptosis^65^.

The low toxicity of PANI/Ag (AMPSA)/GO QDs NC is attributed to the presence of the nontoxic and good environmental stable PANI coating layer of the nanocomposite which decreases the dissolution of the Ag NPs and the release of ions^33,34^. Moreover, The AMPSA capping agent protects and preserves the Ag NPs from the dissolution.

The results regarding exposure of *Artemia salina* to different concentrations of PANI/Ag (AMPSA)/GO QDs are expressed in Table 3. A concentration of 250 mg/L of PANI/Ag (AMPSA)/GO QDs has a negligible acute toxic effect (0% immobilization) for *Artemia salina* after 24 h exposure and 20% immobilization is observed after 48 h. The concentration resulting in 100% immobilization of *Artemia salina* after 48 h is 1000 mg/L. The mean values of EC_50_ and TU for PANI/Ag (AMPSA)/GO QDs after 48 h are 476 mg/L and 0.21, respectively as shown in Table 4.

**Table 3:** *Artemia salina* mean immobilization % after 24 and 48 h of exposure to different concentrations of PANI/Ag (AMPSA)/GO QDs NC.

Concentration (mg/L)	24 h immobilization %	48 h immobilization %
10	0	0
50	0	0
150	0	7
250	0	20
350	10	30
450	20	43
550	40	63
1000	80	100

**Table 4:** EC_50_ and TU of PANI/Ag (AMPSA)/GO QDs NC after 48 h of exposure to *Artemia salina.*

EC_50_	Mean values (mg/L)	Toxicity level	95% confidence limits (mg/L)
EC_50_ at 48 h	476 ± 25.1	Not acutely toxic (EC_50_ ˃100)	121.599–251.037
TU at 48 h	0.21	Low toxic (TU˂1)	

It is found that EC_50_ values of the nanocomposite for the *Ostracods* are lower than that for *Artemia salina*. This is may be due to the high aggregation of PANI/Ag (AMPSA)/GO QDs NC in saltwater to form micro-scale particles. The size distribution of the aggregated nanoparticles is usually not unimodal and the aggregated size increases as the pH approaches the point of zero charge^66^. The pH of PANI/Ag (AMPSA)/GO QDs suspension in the artificial seawater is observed to be 8 ± 0.5, while the point of zero charge of PANI/Ag (AMPSA)/GO QDs NC is at about pH 10.3^32^.

It is reported that nanoparticles suspended in the seawater tend to aggregate in the range from 400 nm up to several microns in diameter^44,67^ and the *Artemia salina* larva are able to ingest them. *Artemia salina* are nonselective filter feeders, and they can readily ingest particles of up to 50 μm in diameter^68^. Several studies have reported that the uptake of nanoparticles by *Artemia* larvae is influenced by the NPs concentration and the time of the exposure while the size of the NPs was not a major factor^44,67^.

### Toxicity of the Cr(VI) treated water

**Figure 1:**
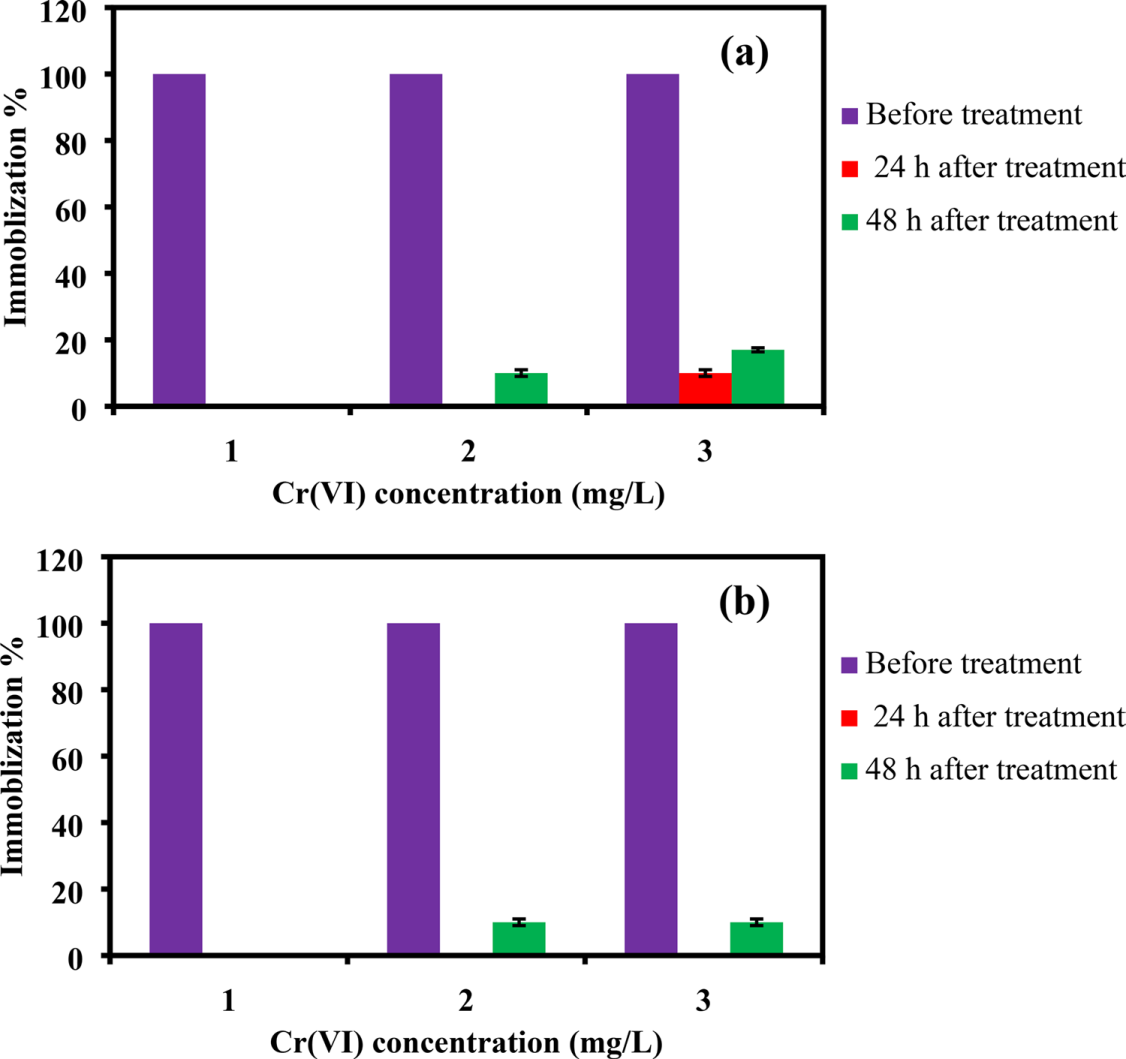
Comparison between the immobilization % of *Ostracods* (**a**) and *Artemia salina* (**b**) larvae exposed to different concentrations of Cr(VI) before and after treatment with PANI/Ag (AMPSA)/GO QDs NC for 24 and 48 h.

In our recent publication^32^, PANI/Ag (AMPSA)/GO QDs NC was applied for the water purification of two water samples containing 60 mg L^−1^ Cr(VI) ions. The results demonstrated that more than 98% of the Cr(VI) ions were removed from the water samples. It was also found that presence of 60 mg L^−1^ of Cr(VI) with multiions did not significantly affect the removal % by PANI/Ag (AMPSA)/GO QDs NC. The toxicity expressed in immobilization % of Cr(VI) solutions with different concentrations of 10, 30, 60 mg/L before and after treatment using PANI/Ag (AMPSA)/GO QDs NC on the *Ostracods* and *Artemia salina* larvae after 24 and 48 h is evaluated as shown in Fig. 1a,b. It is evident that Cr(VI) at these concentrations are completely toxic to both *Ostracods* and *Artemia*. However, using PANI/Ag (AMPSA)/GO QDs NC as Cr(VI) adsorbent plainly caused a significant reduction in the immobilization for the tested concentrations. The NC has reduced the immobilization by 100% for Cr(VI) of 10 mg/L, and 90 and 83% for Cr(VI) of 30 and 60 mg/L, respectively for the *Ostracods* after 48 h exposure as shown in Fig. 1a. For *Artemia*, up to 90% reduction in the immobilization % is observed in 48 h exposure for 60 mg/L Cr(VI) solution after treatment with 1 g/L PANI/Ag (AMPSA)/GO QDs (Fig. 1b). These immobilization reductions are due to the removal of toxic Cr(VI) occurred by the Cr(VI) adsorption onto the NC surface. The chemical interaction between the NC and Cr(VI) is governed by the ion exchange between the dopant (–SO_3_^–^) ions in the adsorbent’s structure and the monovalent bichromate (HCrO_4_^−^) ions in the solutions. Also, the electron donating groups on the NC surface reduce the Cr(VI) ions to the low toxic Cr(III) ions. In addition, the electrostatic attraction can be occurred between the positively charged of polaronic charges of NC and Cr(VI) anions^5,32^.

### Uptake of PANI/Ag (AMPSA)/GO QDs by Ostracods and Artemia salina

**Figure 2:**
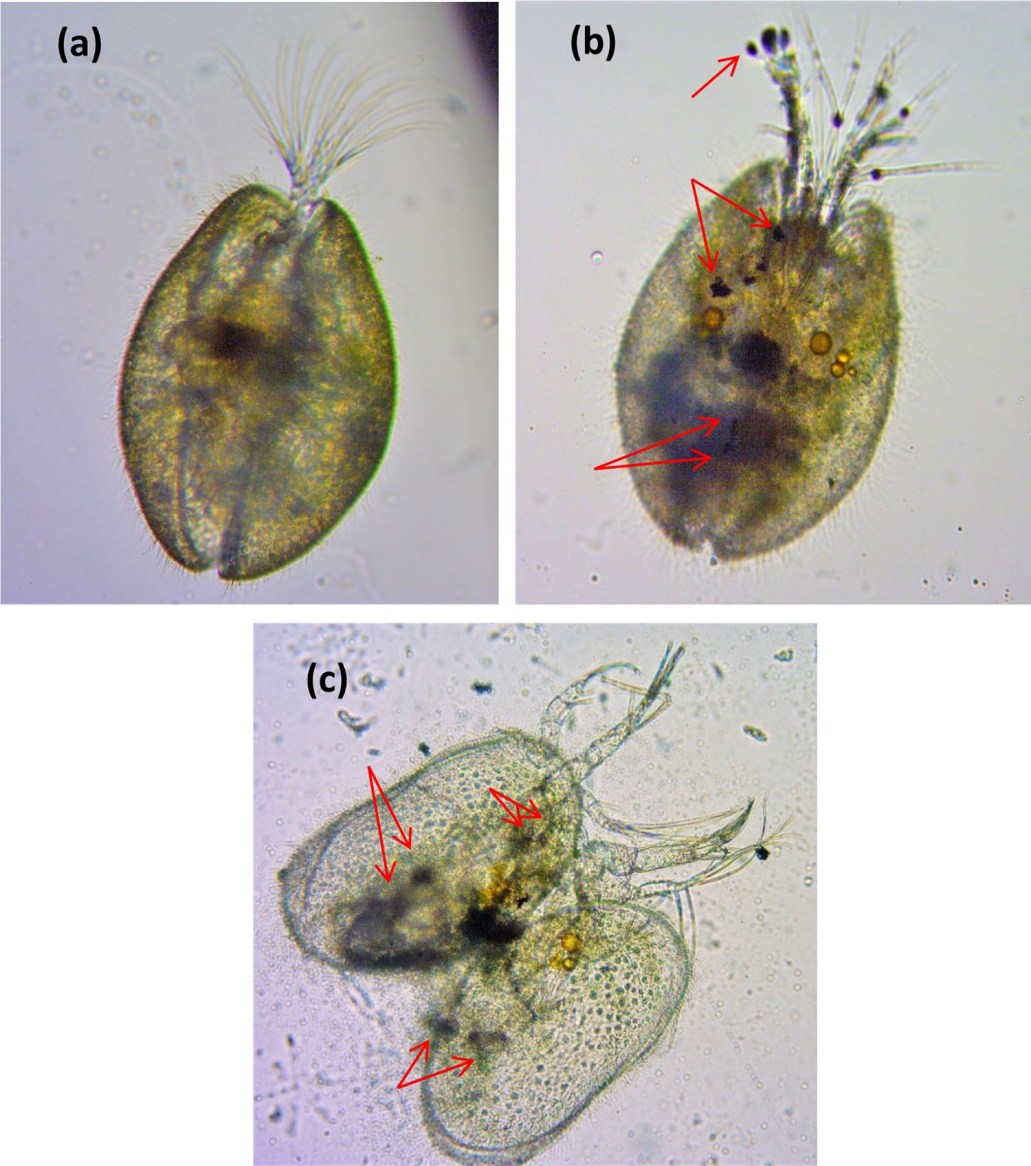
Optical microscope images of the ingestion of PANI/Ag (AMPSA)/GO QDs (red arrows) by *Ostracods* (×40 magnification) after 24 h exposure to 150 mg/L PANI/Ag (AMPSA)/GO QDs. The control is clear of PANI/Ag (AMPSA)/GO QDs (**a**), PANI/Ag (AMPSA)/GO QDs is visible as dark aggregates inside the body of treatment (**b**) and a dead *Ostracod* was ingested PANI/Ag (AMPSA)/GO QDs (**c**).

Organisms used for the acute toxicity tests were collected and observed by an optical microscope to study the nanocomposite ingestion and external deposition. Figure 2 shows changes suffered by *Ostracods* exposed to a solution of 150 mg/L PANI/Ag (AMPSA)/GO QDs NC for 24 h. As can be seen in comparison to the control (Fig. 2a), the dark coloration observed in the *Ostracod* indicates that these organisms ingested the solution of PANI/Ag (AMPSA)/GO QDs NC. It is noted that there are PANI/Ag (AMPSA)/GO QDs agglomerates impregnated in the carapace, antennules and other parts of the body (Fig. 2b). The shells of *Ostracods* are composed of low-magnesium calcite, and in some groups are semi-transparent so that the internal parts can be seen through the carapace^69^. Similar results were reported by using *Heterocypris incongruens Ostracods* as test organism for hazard evaluation of polystyrene nanoplastic and GO, respectively^70,71^. Figure 2c displays the dead *Ostracods* that contains PANI/Ag (AMPSA)/GO QDs aggregates. Dead crustaceans are usually colorless, have their carapaces fully opened, and sometimes they are ripped apart and have their guts spilled over^72^.

**Figure 3:**
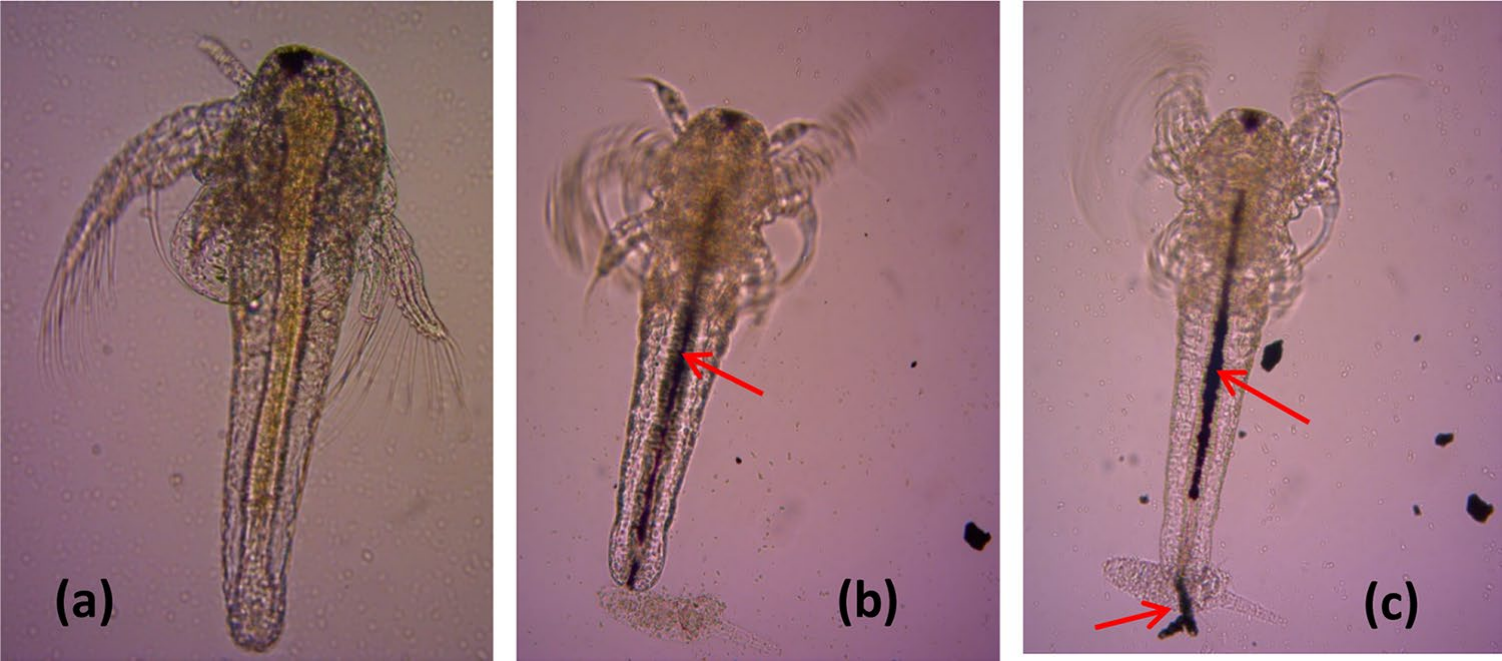
Optical microscope images of the ingestion of PANI/Ag (AMPSA)/GO QDs (red arrows) by *Artemia salina* larvae (×10 magnification) after 24 h exposure to 150 mg/L PANI/Ag (AMPSA)/GO QDs. The gut is empty in the control (**a**), PANI/Ag (AMPSA)/GO QDs is visible as a dark line inside the gut of treatment (**b**) and PANI/Ag (AMPSA)/GO QDs is excreted by *Artemia salina* larvae (**c**).

Ingestion of 150 mg/L PANI/Ag (AMPSA)/GO QDs after 24 h by *Artemia salina* is visually verified also under optical microscope as shown in Fig. 3. The gut is empty in the control (Fig. 3a), after exposure to PANI/Ag (AMPSA)/GO QDs, *Artemia salina* larvae ingest the nanocomposite and the gut is almost entirely filled as manifested by a dark line inside the gut (Fig. 3b). Finally, the accumulated PANI/Ag (AMPSA)/GO QDs NC is excreted by *Artemia salina* (Fig. 3c). The accumulation of NPs inside the gut of *Artemia salina* has already been reported for the ecotoxicity of other NPs^19,44^.

## Conclusions

The acute toxicity effect of PANI/Ag (AMPSA)/GO QDs NC on the aquatic environment was studied using two organisms, freshwater *Ostracods* (*Cypridopsis vidua*) and saltwater *Artemia salina* larvae for 48 h. The mean values of EC_50_ of PANI/Ag (AMPSA)/GO QDs NC powder after 48 h of exposure to the *Ostracods* and *Artemia salina* were 157.6 ± 6.4 and 476 ± 25.1 mg/L, respectively. PANI/Ag (AMPSA)/GO QDs NC was found to be not acutely toxic for both organisms (EC_50_ ˃100), although the nanocomposite was accumulated inside the organisms. The *Ostracods* were appeared to be more sensitive towards PANI/Ag (AMPSA)/GO QDs than *Artemia salina*. It is recommended to fabricate this NC as a filter and exposed the polluted water for this filter to control the contamination of the NC and the adsorbed pollutants.

## Data Availability

All data generated or analysed during this study are included in this published article.
